# A Pseudopeptide Polymer Micelle Used for Asymmetric Catalysis of the Aldol Reaction in Water

**DOI:** 10.3390/polym10091004

**Published:** 2018-09-10

**Authors:** Keyuan Liu, Long Ye, Yao Wang, Ganhong Du, Liming Jiang

**Affiliations:** MOE Key Laboratory of Macromolecular Synthesis and Functionalization, Department of Polymer Science and Engineering, Zhejiang University, Hangzhou 310027, China; lkychem@zju.edu.cn (K.L.); 21529010@zju.edu.cn (L.Y.); 21329021@zju.edu.cn (Y.W.); duganhong@zju.edu.cn (G.D.)

**Keywords:** organocatalysis, micelle, poly(2-oxazoline), asymmetric aldol reaction, pseudopeptide

## Abstract

Micelles assembled from amphiphilic molecules have proved to be ideal scaffolds to construct artificial catalysts mimicking enzymatic catalytic behavior. In this paper, we describe the synthesis of amphiphilic poly(2-oxazoline) derivatives with l-prolinamide units in the side chain and their application in asymmetric aldol reactions. Upon dissolution in water, the pseudopeptide polymers self-assembled into particles with different sizes, relying on the copolymer composition and distribution of hydrophilic/hydrophobic segments in the polymer chain. A preliminary study has demonstrated that the catalytic activity of these polymeric organocatalysts are strongly dependent on the aggregated architecture. The micelle-type assemblies can act as nanoreactors to efficiently promote the direct aldolisation of cyclohexanone with aromatic aldehydes in aqueous media, affording anti-aldol products in excellent yields (88–99%) and higher stereoselectivities (90/10 *dr*, 86% *ee*) compared to their nonmicellar systems under identical conditions.

## 1. Introduction

Since the revival of interest in organocatalysis, the polymeric immobilization of organic catalysts has received considerable research attention as a promising strategy for mimicking enzymatic systems [1,2,3,4,5]. Among the various polymer carriers for loading organocatalysts, the amphiphilic block copolymers are especially attractive as a result of their unique solution properties to associate in both organic and aqueous media to form a range of supramolecular assemblies [6]. By tethering a sort of catalytic entity in the hydrophobic block of the copolymer, the water-compatible catalyst could show a significant improvement in catalytic efficiency by forming specific structures such as micelles. On the other hand, although micellar catalysis in aqueous solution has been known for a long time, polymer-based micelles usually exhibit many advantages over their low-molecular-weight counterparts with respect to structural variability, stability, and functionality [7].

l-Proline and its derivatives, representing the simplest chemical constituents found in type I aldolase enzyme [8], seem to be particularly ideal candidates for the biomimetic design of organocatalysts [9,10,11,12,13,14]. In this regard, an interesting work recently reported by O’Reilly et al. is the development of a kind of proline-functionalized core–shell micelle for aldol reactions in water [15]. The polymeric nanoparticles showed higher catalytic activity compared to non-supported l-proline, which may be ascribed to the ability of the nanostructures to effectively concentrate the reactants in the catalytically active interior. Based on the thermoresponsive property of multiblock copolymers, they also designed more sophisticated nanoreactors for aqueous asymmetric transformation [16]. In this case, the assembly/disassembly process can be cycled repeatedly just by adjusting temperature below or above the lower critical solution temperature of the shell-forming polymer, which greatly facilitates catalyst recovery and recycling together with the isolation of pure product. Despite of remarkable advances achieved in the development of water-compatible polymer-supported organocatalysts for aldol reactions [9,10,11], efficient biomimetic catalytic systems reported thus far remain quite limited. In these catalytic systems, the polymer matrices are mostly confined to styrenics and acrylics [15,16,17,18,19,20]. Such a polymeric catalyst promoting the asymmetric transformation, although highly desired, may be difficult to mimic an enzymatic environment.

Poly(2-oxazoline)s (POXs) are regarded as analogues of polypeptides because of the intrinsic correlation between the two polymer skeletons, which may have potential for biomedicine-related applications [21,22,23]. Given their desirable characteristics, such as a great deal of flexibility within molecular design and chemical stability, we recently explored the feasibility of using the unique tertiary amide motif of poly(2-oxazoline)s to covalently immobilize l-proline catalysts. The resultant l-prolinamido-functionalized polymers have proven to be significantly more effective than their monomeric counterparts for the aldolisation of cyclic ketones with several substituted benzaldehydes under neat conditions, indicating that the polymer backbone played an active and synergistic role in the asymmetric transformation mediated by these species [24]. Along with our ongoing interest in the development of pseudopeptide organocatalysts [24,25], in this work, we designed corresponding amphiphilic copolymer versions with the aim of searching for supramolecular micellar catalysis that more closely resembles enzymatic systems. This paper describes the synthesis of both blocky and random copolymers as well as their self-assembly behavior. As a proof-of-concept application, the as-prepared amphiphilic copolymers were used as an organocatalyst for aldol reactions in aqueous media.

## 2. Materials and Methods

### 2.1. Raw Materials and Instruments

Boc-l-phenylalanine, Boc-l-proline, 2-chloroethylamine hydrochloride, and *O*-(1*H*-benzotriazol-1-yl)-*N*,*N*,*N’*,*N*’-tetramethyluronium tetrafluoroborate (TBTU) were purchased from Energy Chemical (Shanghai, China). 2-Ethyl-2-oxazoline (EtOx) was dried and vacuum distilled over barium oxide before use. Dichloromethane (DCM), triethylamine (Et_3_N), and dimethyl sulfoxide (DMSO) were distilled from calcium hydride. Piperidine was dried by sodium hydroxide and distilled to use. Scandium triflate was prepared according to the reported method [26] and stored in a sealed tube. Other chemicals were obtained from Sinopharm Chemical Reagent Co., Ltd. (Shanghai, China) and used as received.

^1^H NMR spectra were recorded on a Bruker Advance DMX400 spectrometer (Bruker Co., Ltd., Karlsruhe, German) with CDCl_3_ or DMSO-*d*_6_ as the solvent. Chemical shifts were reported in ppm relative to tetramethylsilane (TMS). D-line specific optical rotations ([*α*${]}_{D}^{20}$) were measured in methanol (10 mg/mL) at 20 °C using a Perkin-Elmer 341 LC polarimeter (Perkin-Elmer Co., Ltd., Waltham, MA, USA). Size exclusion chromatography (SEC) analyses were carried out on a Waters 150C apparatus (Waters Co., Ltd., Milford, MA, USA) equipped with two PLgel 5-μm MIXED-C (300 mm × 7.5 mm) columns (for DMF phase) or Styragel Columns HR4, HR3, and HR1 (for THF phase) and a differential refractometer detector using DMF (flow rate: 1 mL/min, 60 °C) or THF (flow rate: 1 mL/min, 40 °C) as the eluent, respectively. The number-average molecular weight (*M*_n_) and polydispersity index (*Đ*) of the polymers were calculated based on PMMA or polystyrene (PS) calibration. High performance liquid chromatography (HPLC) analyses were performed on a Chromeleon apparatus (Dionex Co., Ltd., Sunnyvale, CA, US) equipped with chiral columns (Daicel Chiralpak AD-H, 4.6 mm × 250 mm) employing *n*-hexane/*i-*PrOH (90/10, *v*/*v*) as the eluent (flow rate: 0.8 mL/min) and UV detection (*λ* = 254 nm). Dynamic light scattering (DLS) was performed on a Zetasizer Nano Series (Malvern Instruments, Malvern, UK) at 657 nm and a detection angle of 173°. Transmission electron microscopy (TEM) images were captured on a Hitachi HT7700 electron microscope system (Hitachi Co., Ltd., Tokyo, Japan) at an acceleration voltage of 100 kV. An ImageJ software (v 1.51, National Institutes of Health, Bethesda, MD, USA) was used to label the diameters of 100 randomly selected micelles, and the results are expressed in the form mean ± standard deviation.

### 2.2. Synthesis of Polymeric Catalysts

**Scheme 1:**
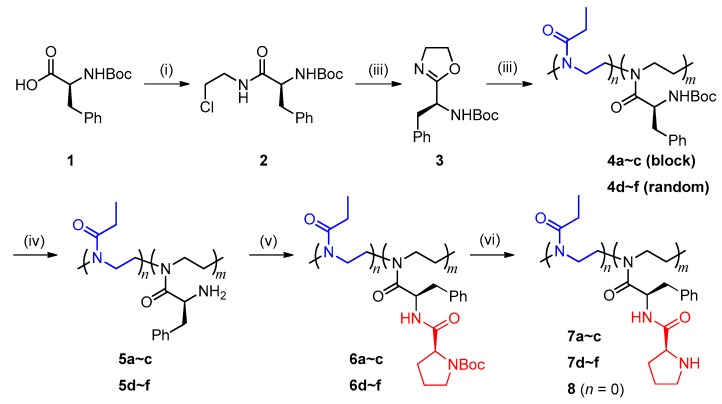
Synthesis of L-prolinamido-poly(2-oxazoline)s. (i) ClCH_2_CH_2_NH_2_·HCl, TBTU, Et_3_N, DCM, 0 °C~r.t.; (ii) NaOH, MeOH, 40 °C; (iii) EtOx, Sc(OTf)_3_, CH_3_CN, 90 °C; (iv) TFA, DCM, r.t.; (v) Boc-L-proline, TBTU, Et_3_N, DMSO, r.t.; (vi) TFA, DCM, r.t.

The synthesis of poly(2-oxazoline)-bound l-prolinamide catalysts (l-prolinamido-POXs) is shown in Scheme 1, including synthesis of the functional monomer (*S*)-2-(1-Boc-amino-2-phenyl)ethyl-2-oxazoline **3** (i–iii), cationic ring-opening copolymerization of **3** with EtOx (iv), and post-modifications towards the precursor polymer **4** (v–vi). 

#### 2.2.1. *(S)*-2-(1-Boc-amino-2-phenyl)ethyl-2-oxazoline (**3**)

The monomer was prepared from Boc-l-phenylalanine via two-step reactions in high yield according to the reported method [27]. Boc-l-phenylalanine (20.0 g, 75.4 mmol), 2-chloroethylamine hydrochloride (9.6 g, 82.9 mmol), and TBTU (26.6 g, 82.9 mmol) were dissolved in DCM (200 mL) followed by cooling to 0 °C. Trimethylamine (31.5 mL, 226.2 mmol) was added to the solution dropwise. The mixture was stirred overnight. After removal of the solvent, the resulted residue was purified by silica gel column chromatography with ethyl acetate/petroleum ether (1/2, *v*/*v*) as the eluent to give product **2** as white solid (22.4 g, 91%).

**2** (20.0 g, 61.2 mmol) was added to methanol solution of NaOH (7.3 g, 183.6 mmol, 250 mL). After stirring for 32 h at 40 °C, the solvent was removed under reduced pressure. The residue was dissolved in DCM (200 mL), washed with deionized (DI) water (200 mL × 3), dried over Na_2_SO_4_, filtered, and concentrated under reduced pressure to afford crude product (14.9 g, 84%). Finally, the product (**3**) was purified by recrystallization from EtOAc/hexane. M.p. 128.5~129.6 °C; [*α*${]}_{D}^{20}$ = +8° (10 mg/mL, MeOH); ^1^H NMR (400 MHz, CDCl_3_, *δ*/ppm): 7.36~7.05 (m, 5H), 5.10 (d, 1H), 4.67 (d, 1H), 4.22~4.36 (m, 2H), 3.88~3.69 (m, 2H), 3.09 (ddd, 2H), 1.41 (s, 9H); ^13^C NMR (100 MHz, CDCl_3_, *δ*/ppm): 167.09 (s), 154.90 (s), 136.31 (s), 129.50 (s), 128.30 (s), 126.79 (s), 79.66 (s), 68.11 (s), 54.09 (s), 49.74 (s), 38.78 (s), 28.31 (s); ESI-MS: *m*/*z* (%) = 290.9 (8.8) [M + H]^+^, 312.9 (10) [M + Na]^+^, 602.9 (100) [2M + Na]^+^. See: Figures S1–S3 in the Supplementary Materials (SM) for NMR and ESI-MS spectra.

#### 2.2.2. Cationic Ring-Opening Copolymerization of Monomer **3** and 2-ethyl-2-oxazoline (EtOx)

The general procedure for block-copolymerization (where [3]/[EtOx]/[**I**] = 100/100/1) is described as follows. To a dried flask (Synthware Co., Ltd., Beijing, China) was added monomer **3** (1.20 g, 4.1 mmol) under nitrogen atmosphere. A solution of Sc(OTf)_3_ (2.07 mL, 0.041 mmol) in CH_3_CN was added as the initiator. The mixture was heated to 90 °C, stirred for 2 h, and then EtOx was added as the comonomer (0.42 mL, 4.1 mmol). The polymerization was conducted for a further 2 h and terminated by the addition of piperidine (0.10 mL, 1.2 mmol). The resulting solution was concentrated under reduced pressure, dissolved in THF (4 mL), and precipitated out in diethyl ether/*n*-hexane (1/3, *v/v*, 100 mL). The crude product was purified by several dissolution-precipitation processes to give the desired polymer **4b** as a white solid (1.35 g, 83%). The random copolymers were prepared by the same method, in which the monomer mixture was fed at one time.

#### 2.2.3. Postmodification of Precursor Polymers 

To the mixture of TFA/DCM (1/1, *v/v*, 24 mL) was added **4** (1.20 g, 3.1 mmol) and the resulting solution was stirred for 24 h at room temperature. After removal of the solvent, the residue was dissolved in MeOH (4 mL) and neutralized with saturated NaHCO_3_ solution, followed by dialysis against deionized water (*M*_w_ cut-off 1000) and lyophilization to afford the deprotected product **5** as a white solid (0.84 g, 90%).

**5** (0.70 g, 2.4 mmol), Boc-l-proline (0.63 g, 2.9 mmol), and TBTU (0.93 g, 2.9 mmol) were dissolved in 200 mL DCM and cooled with an ice bath. To the resulting solution was added Et_3_N (1.0 mL, 7.2 mmol) dropwise and then stirred for 48 h. The mixture was extracted with deionized water (200 mL × 3) and the collected organic phase was concentrated under reduced pressure. The resulting solid was dissolved in a minimum amount of DMF, and an excess amount of hexane was added to precipitate out the desired product **6** (1.1 g, 93%).

**6** (1.00 g, 2.0 mmol) was added into the mixture of TFA/DCM (1/1, *v/v*, 20 mL) and stirred for 24 h at room temperature. After removal of the solvent, the residue was dissolved in MeOH (4 mL) and neutralized with saturated NaHCO_3_ solution, followed by dialysis against deionized water (*M*_w_ cut-off 1000) and lyophilization to afford the target catalyst **7** as a white solid (0.87 g, 87%).

### 2.3. Micellization of Amphiphilic Copolymers

Polymer micelles were prepared by dialysis according to a generally adapted method [28]. A THF solution of **7** (10 mg/mL) was prepared and dialyzed against deionized water using a dialysis membrane tube (Yuanye biotechnology Co., Ltd., Shanghai, China) with a *M*_w_ cut-off of 3500 for 2 days at room temperature. The aqueous micelle solutions were directly used for DLS and TEM measurements and catalytic reactions.

### 2.4. General Procedure for Aldol Reaction

Direct asymmetric aldol reaction of cyclohexanone and *p*-nitrobenzaldehyde was selected as the model reaction. The reactions were carried out under unified and optimized conditions according to our previous studies [24]. To an aqueous solution of catalyst (37.5 μmol in 1 mL DI water, loading 15 mol % of proline groups relative to the aldehyde) was added *p*-nitrobenzaldehyde (0.25 mmol) and cyclohexanone (5 mmol) with rapid stirring to afford emulsion. TFA (1 μL) was added in contrast experiments. The reaction was carried out at 10 °C and monitored by TLC analysis until reaction completion. The mixture was then extracted with ethyl acetate three times. The collected organic phase was dried over Na_2_SO_4_, filtered, and concentrated under reduced pressure. Purification by column chromatography using ethyl acetate and petroleum ether (1/4, *v/v*) as the eluent resulted the aldol product as a pale yellow powder.

## 3. Results and Discussion

### 3.1. Polymerization and Structural Characterization of Polymers

The synthesis of well-defined copolymers **4** is a key step in the preparation of polymeric catalysts. According to our previously reported procedure [29], the ring-opening copolymerization of **3** with EtOx was allowed to occur in a random or sequential fashion in CH_3_CN using Lewis acidic Sc(OTf)_3_ as the initiator.

Table 1 summarizes the polymerization results as well as the copolymer composition, yield, the molar mass, and its polydispersity index (*Đ*). Under optimal conditions, copolymers having a controlled molecular weight and relatively narrow polydispersity (*Đ* = 1.33–1.45) could be obtained in moderate to high yield. However, the yield decreased by increasing the content of **3** in the monomer feed in either sequential or random copolymerization (entries 1–3 and 4–6). Meanwhile, the copolymer composition deviated slightly from the theoretical values at a higher monomer/initiator ratio. In all the experiments, SEC analyses gave unimodal molar mass distributions for the resultant polymers (Figure S4 in SM). The ring-opening copolymerization was achieved in a controllable fashion, as previously reported [24,28,29].

**Figure 1:**
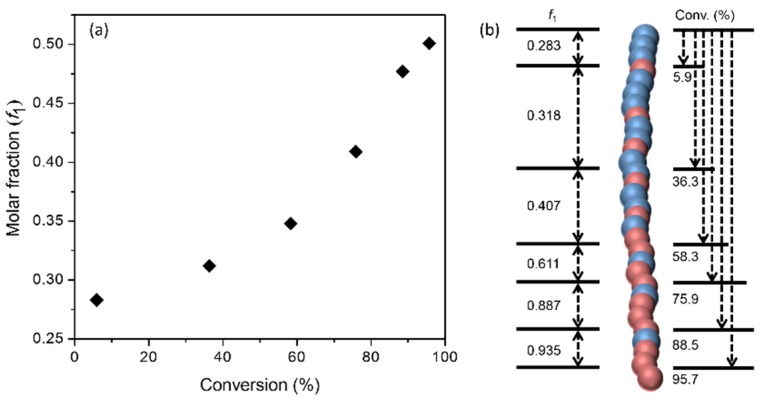
(**a**) Molar fraction (*f*_1_) of **3** in the copolymers vs. monomer conversion and (**b**) schematic representation of gradient polymer structures. Copolymerization: [**I**]:[3]:[EtOx] = 1:50:50; the monomer conversion was monitored with ^1^H NMR spectroscopy.

On the other hand, the monomer reactivity ratios of **3** and EtOx were determined to be 0.044 and 2.832, respectively. The measurements were conducted according to the Fineman–Ross (FR) method [30] (see Figure S5 and Table S1 in SM). Apparently, regarding the reactivity of both monomers, it is justified to reason that their polymerization proceeds with a much different rate. Figure 1 plots, as an example, the changes in molar fraction (*f*_1_) of **3** in the copolymers as a function of monomer conversion, which clearly demonstrated the formation of gradient copolymers instead of strictly statistic copolymers in the abovementioned random copolymerization. This was also confirmed indirectly by their self-assembly behavior in water (vide infra).

### 3.2. Postmodification of Copolymer **4**

**Figure 2:**
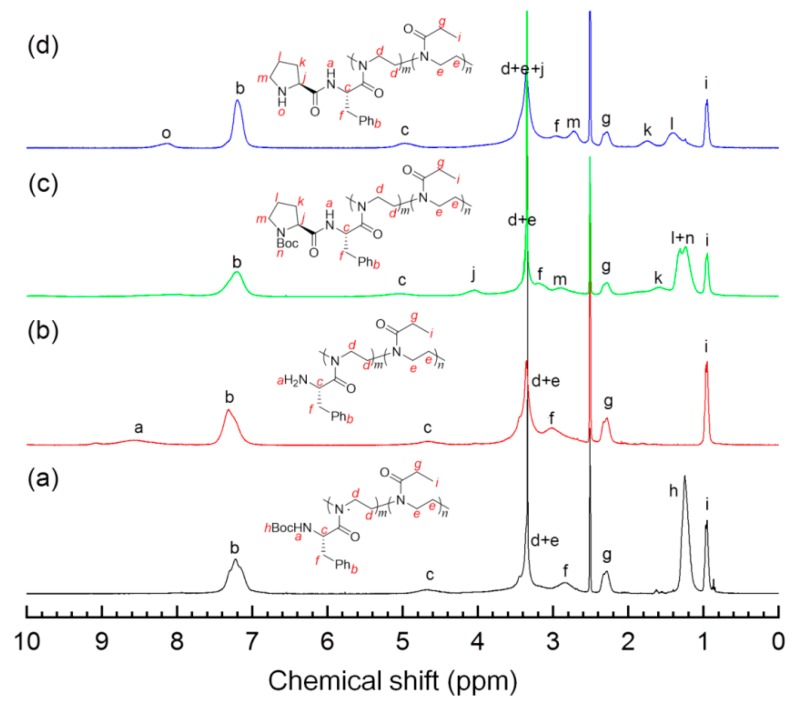
^1^H NMR spectra (DMSO-*d*_6_, 400 MHz) of the copolymers collected at different reaction stages: (**a**) **4b**, (**b**) **5b**, (**c**) **6b**, and (**d**) **7b**.

^1^H NMR spectroscopy was employed to track the postmodification process of the precursor polymers **4**. As an example, Figure 2a,d compares ^1^H NMR spectra of the polymer **4b** and its derivatives **5b**, **6b**, and **7b**, all of which show the characteristic signals of the methane proton (*d–e*) of the main chains around 3.4 ppm. The signal of Boc groups, observed for **4b** in Figure 2a at 1.25 ppm, disappeared fully in the spectrum of **5b** (Figure 2b), and a weak band (*a*) centered at ~8.6 ppm from the protonated amine groups concomitantly appeared, indicating that deprotection was achieved. In Figure 2c, **6b** showed ^1^H NMR signals arising from pyrrolidinyl moieties at 4.0 (*j*), 2.0–1.5 (*k*, *l*) and ~2.8 ppm (*m*) as well as those due to Boc protons at 1.44–1.06 ppm (*n*), which confirm the cooperation of the prolinamide moieties. Calculated from the relative integration values, i.e., “*n*” vs. “*i*” in Figure 2c and “*h*” vs. “*i*” in Figure 2a, the *L*-proline functionalization (the grafting efficiency) was estimated to be more than 90% in the preparation of **6a**–**e** (see Table 2). As evidenced by Figure 2d from the absence of a peak at ~1.25 ppm that is characteristic of Boc groups, the deprotection was almost quantitatively completed, giving the corresponding polymeric catalysts with different unit ratios in good yields (>85%). It is worth mentioning that the signal from amide N–H was not detected as a consequence of the proton exchange with trace water in the deuterated solvent [31,32].

Another point which should be noted is that the polymeric catalysts **7** were not suitable for SEC measurements due to their strong interaction with column fillers [28]. Therefore, the molecular weights of **7** were indirectly characterized in their Boc-protected form (i.e., **6**). As seen from Table 2, all the *N*-Boc-prolinamide derivatives except for **6f** showed lower *M*_n_ values than the corresponding precursors (**4**) of the same copolymer composition when compared pairwise, which might be attributed to their different hydrodynamic properties. However, the polydispersity indices of the two were similar.

### 3.3. Self-Assembly Behavior of Polymer **7** in Water

**Figure 3:**
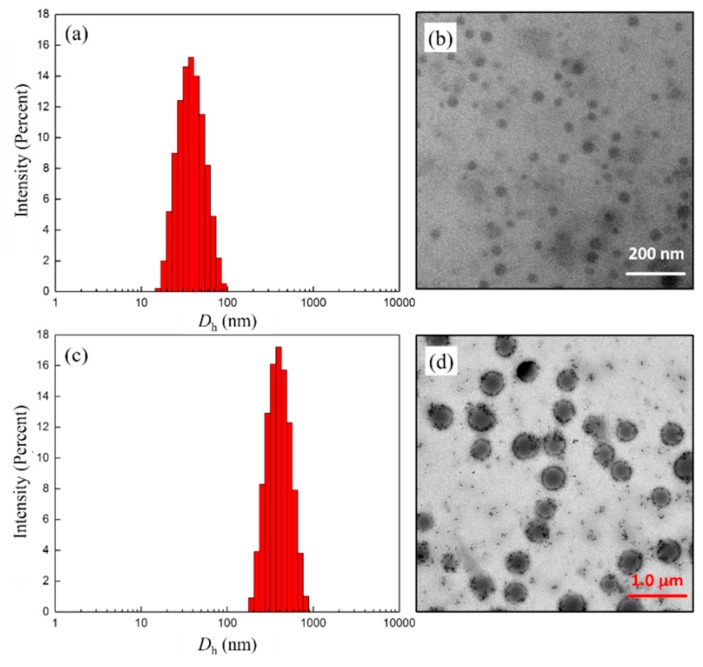
DLS results and TEM images obtained from aqueous solutions of copolymers **7c** (**a**,**b**) and **7f** (**c**,**d**), respectively. Sample conc. = 1 mg/mL.

After removing the Boc-group from **6**, all the deprotected products (copolymers **7**) showed good water solubility, and the Tyndall effect could be observed in the aqueous solution. The combination of DLS and TEM measurements on these systems revealed that the colloidal dispersions consisted of particles of tens to hundreds of nanometers, depending on the ratio of hydrophilic/hydrophobic segments and their distribution in the polymer chain. For example, from Figure 3a,c the intensity-average hydrodynamic diameter (*D*_h_) was 35 nm and 304 nm for the block copolymer **7c** and its random counterpart **7f**, respectively. Correspondingly, the TEM images showed the present morphologies of presumably spherical aggregates with diameters of about 30 and 310 nm. Taking into account both the hydration degree and the polydispersity effects, the mean particle sizes estimated by TEM compared reasonably well to the average *D*_h_s. It is worth noting, however, that the gradient copolymer **7f** seemed to self-assemble into large compound aggregates induced by inter/intrapolymer hydrogen bonding, which is similar to those reported in the literature [33,34]. Furthermore, for the polymer **7f**, the lack of a suitable hydrophilic–hydrophobic balance most likely led to the formation of a large number of small-sized assemblies during dialysis. From TEM observations of **7f** self-assembling in THF/H_2_O (Figure 3d), these small sphere-like particles adhere to the surface of large aggregates or exist in a free state.

For the block copolymers, the average *D*_h_ value undergoes a significant decrease from 212 (**7a**) to 35 nm (**7c**) when the molar ratio of the hydrophobic segment increases from 25% to 80%, which is in line with the general rule of amphiphilic assemblies [6]. In contrast, the aggregates formed from the gradient copolymers **7d**–**f** showed much larger *D*_h_s (304–426 nm) than that of the corresponding block copolymers with similar composition and grafting percentage (see Table 3, **7a** vs. **7d**, **7b** vs. **7e**, and **7c** vs. **7f**). This result indicated that the hydrophilic/hydrophobic ratio and its distribution in the main chain played a key role in the formation of self-assembling nanostructures. In fact, the amphiphilic block copolymers (**7a**) with high EtOx content (>50 mol%) exhibited a weak tendency to self-assemble, so it was hard to get high-quality TEM images for its aqueous solution (see Figure S6). As for the homopolymer **8**, it was not suited for water-phase DLS and TEM measurements due to its hydrophobicity.

### 3.4. Evaluation of Catalytic Activity

The aldol reaction of *p*-nitrobenzaldehyde with cyclohexanone was employed as a benchmark test of the polymeric catalysts (Table 4), as frequently reported in the literature [35,36,37,38]. In the absence of an acidic additive, the reaction proceeded smoothly in water, affording aldol products in 24 h with excellent yield (85–96%) and moderate to good diastereoselectivity (71:29–90:10 anti:syn ratio). Comparable enantioselectivity was observed for the antiproduct in the reactions using the blocky and random copolymer catalysts (58–70% *ee*) with the exception of **7d** (31% *ee*, entry 7). Upon addition of TFA, which has often been used as an acidic additive to improve catalytic efficiency in many organocatalytic transformations [39,40,41], all catalysts gave aldol products in better yield (90–99%) with higher enantioselectivities ranging from 71% to 86%. More importantly, the *ee* value improved from 71% for the block copolymer **7a** to 77% for **7b** and to 86% for **7c** (entries 2, 4, and 6). In other words, the trend of enantioselectivity enhancement corresponded well to a hydrodynamic diameter decrease from 212 to 35 nm (entries 1–3 in Table 3). A similar tendency in enantioselectivity was observed in the case without TFA (entries 1, 3, and 5 in Table 4). However, the random copolymers, or more strictly, gradient copolymers **7d**–**f**, did not seem to follow this general rule. In this case, the *ee* values were almost the same (80–82%) for the copolymers with different hydrophilic/hydrophobic ratios, although a higher enantioselectivity was achieved with TFA compared to the systems without the acidic additive (entries 7–12).

Overall, the stereoselectivity of the block copolymer **7c** was superior to that of **7f** and the homopolymer **8** and their activities were comparable (see entries 6, 13, and 14 in Table 4). The higher catalytic efficiency of **7c** with respect to its analogues (**7a**, **7b**, or **7d**–**f**) might be explained by the relatively small particle size of ~30 nm. The polymer chains tucked in the micellar interior was expected to establish a microenvironment favorable for strengthening the interaction between the catalytic cites and reactants, thus leading to a higher catalytic activity and to higher levels of stereocontrol in the aqueous media [42]. As demonstrated in light scattering experiments, upon introduction of reactants, an increase in micelle sizes was observed, suggesting that the hydrophobic substrates are concentrated in the catalytically active inner core, and the nanostructure was not destroyed even in the case of TFA being added (Figure S7).

## 4. Conclusions

In summary, a class of amphiphilic poly(2-oxazoline) derivatives bearing a l-prolinamide pendant was synthesized by the combination of controlled cationic ring-opening polymerization and a postmodification strategy. Upon direct dissolution in water, the copolymers self-assembled into micelle-type aggregates with particle sizes of tens to hundreds of nanometers depending on the different hydrophilic/hydrophobic ratios and their distribution in the main chain. In the aldol reaction of cyclohexanone with *p*-nitrobenzaldehyde proceeded in aqueous media, the block copolymer catalyst **7c** gave optimal results in terms of both the yield (up to 99%) and stereoselectivity (90/10 *dr*, 86% *ee*), pointing to the existence of a fortunate micellar effect. Although the polymer-based micellar catalyst presents some limitations and more work remains to be done to improve activity and stereoselectivity, this study provides a new design strategy based on the pseudopeptide scaffolds for biomimetic catalytic systems.

## Figures and Tables

**Table 1:** Copolymerization results of **3** with 2-ethyl-2-oxazoline (EtOx) initiated with Sc(OTf)_3_^1^.

Entry	Feed Ratio [3]/[EtOx]/[I]	Poly-3		Copolymer 4				
		*M*_n_^2^	*Đ* ^2^	Code	*M*_n_^2^	*Đ* ^2^	Yield (%) ^3^	3 (mol%) ^4^
1	50/150/1	4900	1.19	**4a**	8700	1.33	90	25
2	100/100/1	7800	1.25	**4b**	11,400	1.38	83	50
3	150/50/1	11,600	1.34	**4c**	14,000	1.45	60	80
4	50/150/1	-	-	**4d**	9900	1.34	96	25
5	100/100/1	-	-	**4e**	10,700	1.37	91	47
6	150/50/1	-	-	**4f**	10,800	1.33	80	71

^1^ [M] = 2 mol/L, 90 °C; Entries 1–3 and 4–6 for block and random copolymerization, respectively; see: Experimental section. ^2^ Determined by SEC with PMMA calibration, DMF as the eluent; *Đ* = *M*_w_/*M*_n_. ^3^ Isolated yield. ^4^ Calculated by ^1^H NMR (CDCl_3_).

**Table 2:** SEC characterization results of the copolymer **6.**

Entry	Code	*M*_n_^1^	*Đ* ^1^	*E*_f_ (%) ^2^
1	**6a**	6600	1.30	98
2	**6b**	8700	1.39	97
3	**6c**	10,000	1.54	90
4	**6d**	8200	1.23	97
5	**6e**	9600	1.27	95
6	**6f**	11,900	1.30	91

^1^ PMMA calibration, DMF as the eluent. ^2^ Calculated by ^1^H NMR (CDCl_3_).

**Table 3:** Results of DLS and TEM measurements of **7**.

Entry	Code	DLS		TEM
		*D*_h_ (nm) ^1^	PDI ^1^	Size (nm) ^2^
1	**7a**	212	0.259	190 ± 60
2	**7b**	85	0.330	60 ± 20
3	**7c**	35	0.112	30 ± 10
4	**7d**	426	0.198	380 ± 60
5	**7e**	376	0.182	330 ± 60
6	**7f**	304	0.085	310 ± 70

^1^*c* = 1 mg/mL, DI water, 10 °C. ^2^ The statistical size was calculated from TEM images of **7a**–**f** (as shown in Figure 3 and Figure S6). All the samples were prepared from 1 mg/mL micelle aqueous solutions.

**Table 4:**
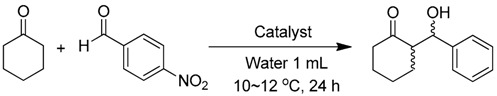
Aldol reactions of cyclohexanone and *p*-nitrobenzaldehyde in water ^1^

Entry	Catalyst	TFA (μL)	Yield (%) ^2^	anti/syn ^3^	*ee* (%) ^4^
1	7a	0	85	86/14	58
2	7a	1	90	83/17	71
3	7b	0	96	87/13	63
4	7b	1	99	88/12	77
5	7c	0	93	87/13	67
6	7c	1	96	90/10	86
7	7d	0	94	71/29	31
8	7d	1	91	81/19	82
9	7e	0	89	86/14	70
10	7e	1	93	87/13	82
11	7f	0	91	77/23	65
12	7f	1	96	83/17	80
13	8 ^5^	0	97	77/23	52
14	**8** ^5^	1	96	81/19	80

^1^ Reaction was performed in a mixture of deionized (DI) water (1 mL), *p*-nitrobenzaldehyde (0.25 mmol), and cyclohexanone (5 mmol) in the presence of 37.5 μmol catalyst (based on the actual concentration of proline groups, 15 mol% relative to the aldehyde). The data in the table are the average of three parallel experiments, and the error is controlled within 5%. ^2^ Isolated yield. ^3^ Diastereomeric ratio, determined by ^1^H NMR (CDCl_3_). ^4^ Determined by HPLC with chiral column (Figure S8); the absolute configuration of the products was deduced by comparing the retention time with reported values [43,44,45]. ^5^ *M*_n_ ≈ 5800, *M*_w_/*M*_n_ = 1.28 [24].

## Supplementary Materials

The following are available online at http://www.mdpi.com/2073-4360/10/9/1004/s1, Figure S1: ^1^H NMR spectrum of **3** (CDCl3, 400 MHz), Figure S2: ^13^C NMR spectrum of **3** (CDCl_3_, 101 MHz), Figure S3: ESI-MS of **3**, Figure S4: SEC curves of diblock copolymers (a) and random copolymers (b), Figure S5: FR plot for the copolymerization of **3** and EtOx at low conversion, Figure S6: TEM images of aqueous solutions of **7a**, **7b**, **7d**, and **7e**; see Table 3, Figure S7: DLS results of **7c** aq. solution (1 mg/mL) before and after addition of (a) cyclohexanone (~0.2 mg/mL) and (b) TFA (1 μL/mL), Figure S8: A representative HPLC analysis of the aldol products. Mobile phase: *n*-hexane: *i*PrOH = 1:9, 0.8 mL/min, Table S1: FR parameters for copolymerization of **3** with EtOx at low conversion ^a^.
