# Supplementary material for: A Pseudopeptide Polymer Micelle Used for Asymmetric Catalysis of the Aldol Reaction in Water
Source: Polymers (Basel). 2018 Sep 10;10(9):1004. doi: 10.3390/polym10091004 (PMC6403597; doi:10.3390/polym10091004)
Supplement: Supplementary file 1 [file polymers-10-01004-s001.pdf]

Supplementary Materials

A Pseudopeptide Polymer Micelle Used for Asymmetric Catalysis of the Aldol Reaction in Water

Keyuan Liu, Long Ye, Yao Wang, Ganhong Du, Liming Jiang *

MOE Key Laboratory of Macromolecular Synthesis and Functionalization; Department of Polymer Science and Engineering; Zhejiang University, Hangzhou 310027, China

* Correspondence: cejlm@zju.edu.cn

1. Structural Characterization of the Monomer 3

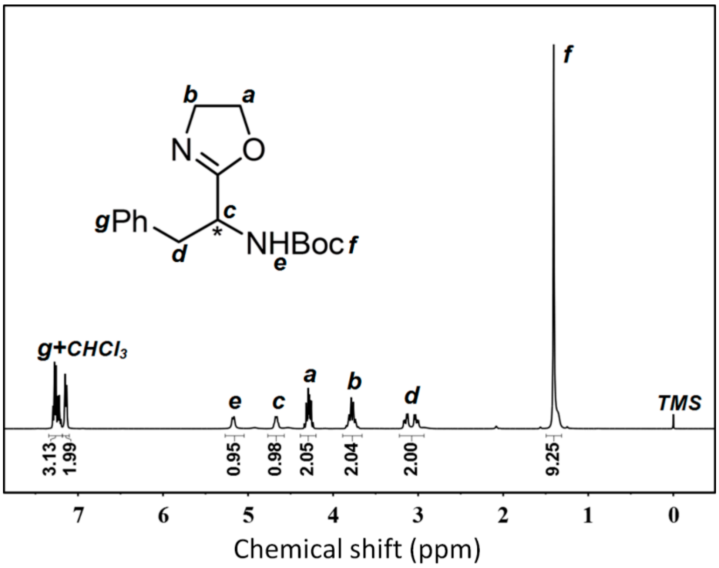

Figure S1. ^{1}H NMR spectrum of **3** ($CDCl_3$, 400 MHz).

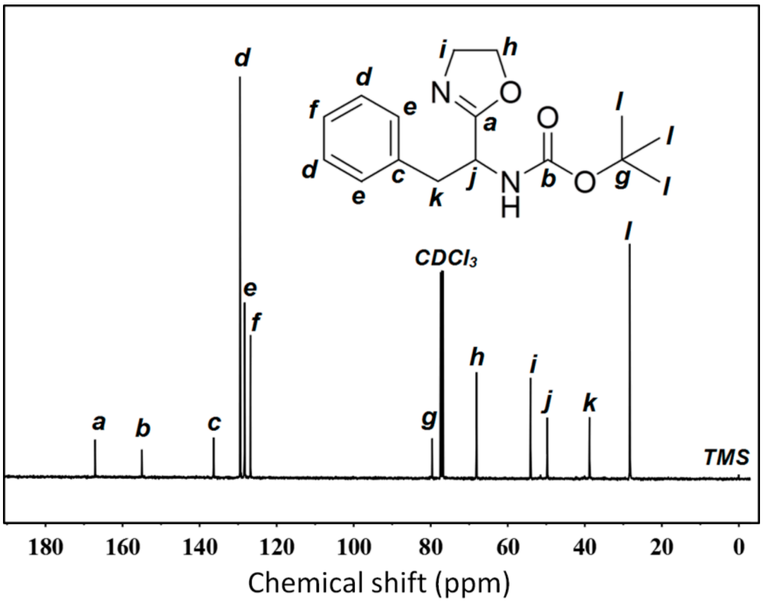

Figure S2. ^{13}C NMR spectrum of **3** ($CDCl_3$, 101 MHz).

Polymers **2018**, *10*, 1004; doi: 10.3390/polym10091004

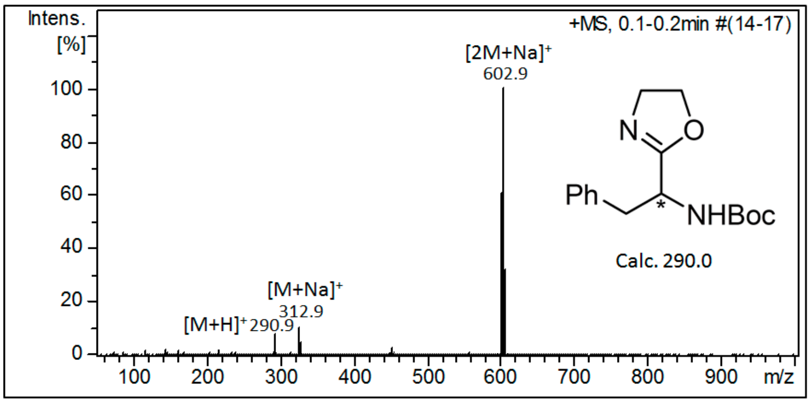

Figure S3. ESI-MS of **3**.

2. SEC Characterization of Copolymers

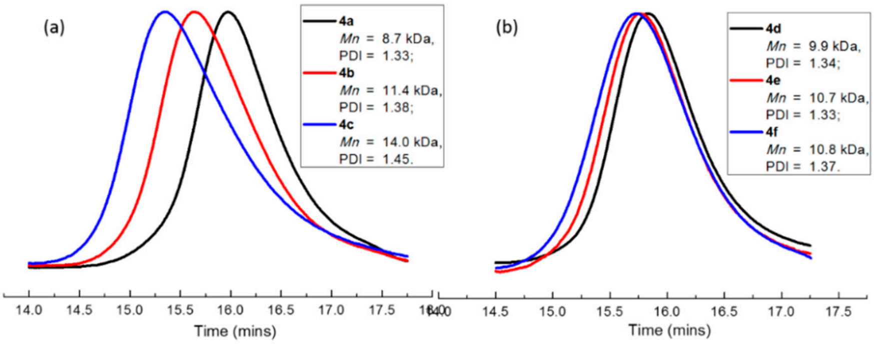

Figure S4. SEC curves of diblock copolymers (**a**) and random copolymers (**b**).

3. Determination of Monomer Reactivity Ratios

Based on the random copolymerization of (*S*)-2-(1-Boc-amino-2-phenyl)ethyl- 2-oxazoline (**3**) and EtOx, the monomer reactivity ratios of **3** and EtOx were determined by linear regression analysis according to the Fineman–Ross (FR) method.[1] The FR equation is given below:

$$\frac{1-f}{F} = \frac{f}{F^2} r_2 - r_1$$

where r_1 and r_2 are the reactivity ratios of **3** and EtOx and F and f represent the feed molar ratio of **3** to EtOx and the corresponding molar ratio of copolymer composition, respectively. The value of $\frac{1-f}{F}$ should be in a linear relationship with $\frac{f}{F^2}$ theoretically. The slope could be calculated as r_2 and the intercept was considered as $-r_1$. Figure S7a shows the FR linear extrapolation plot for copolymerization of **3** and EtOx via the polymerization data listed in Table S1. The reactivity ratios of **3** (r_1) and EtOx (r_2) were calculated to be 0.044 and 2.832, respectively.

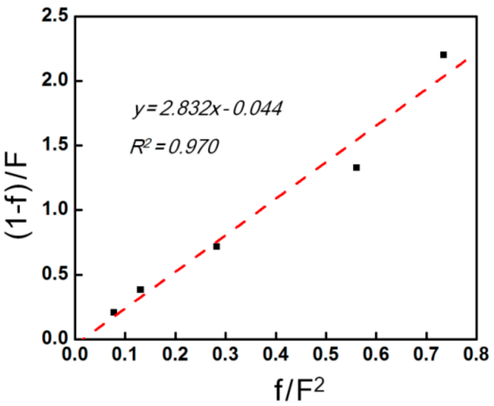

Figure S5. FR plot for the copolymerization of **3** and EtOx at low conversion.

Table S1. FR parameters for copolymerization of **3** with EtOx at low conversion. [a]

Entry	F	f [b]	$\frac{f}{F^2}$	$\frac{1-f}{F}$	Conversion rate (%) [c]
1	2.500	0.483	0.078	0.207	3.2
2	1.670	0.362	0.130	0.383	4.7
3	1.000	0.283	0.283	0.717	5.9
4	0.600	0.202	0.561	1.330	6.6
5	0.400	0.118	0.734	2.202	8.1

[a] Conditions: I = $Sc(OTf)_3$; $[M]_{0,total}/[I]_0$ = 100, [M] = 2 mol/L in CH_3CN, 90 °C. [b] Calculated by ^1H NMR ($CDCl_3$). [c] Isolated yield.

4. DLS and TEM Measurements

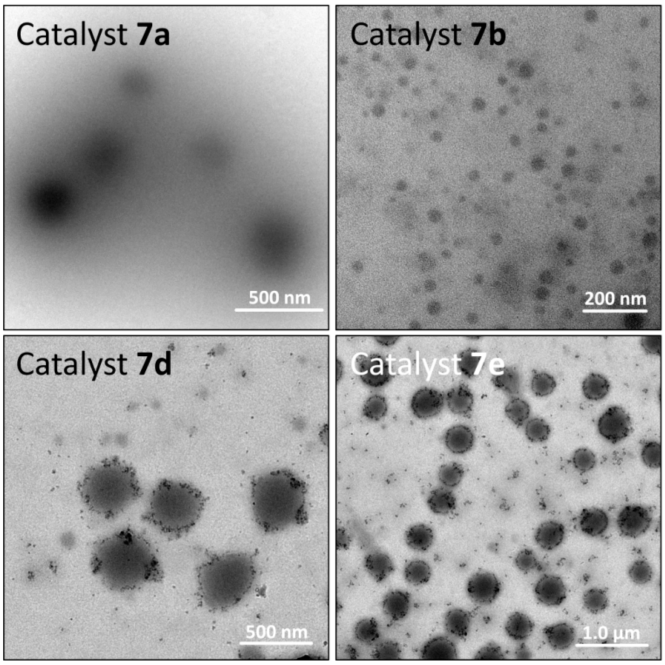

Figure S6. TEM images of aqueous solutions of **7a**, **7b**, **7d**, and **7e**; see Table 3.

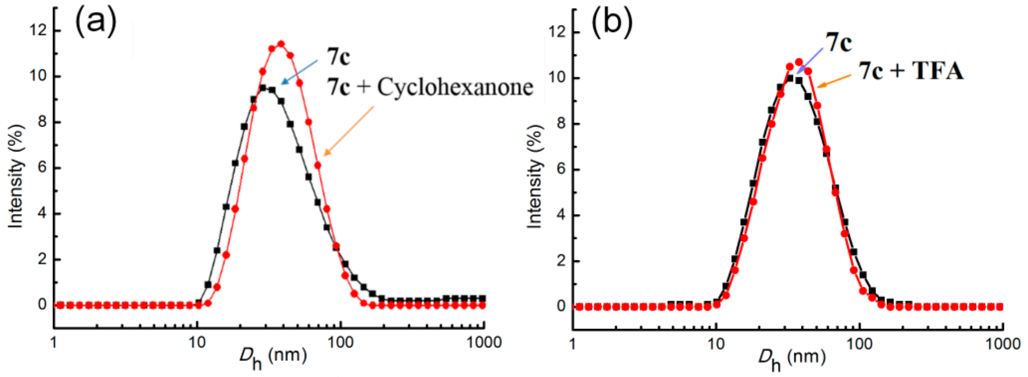

Figure S7. DLS results of **7c** aq. solution (1 mg/mL) before and after addition of (**a**) cyclohexanone (~0.2 mg/mL) and (**b**) TFA (1 μL/mL).

5. HPLC Analysis

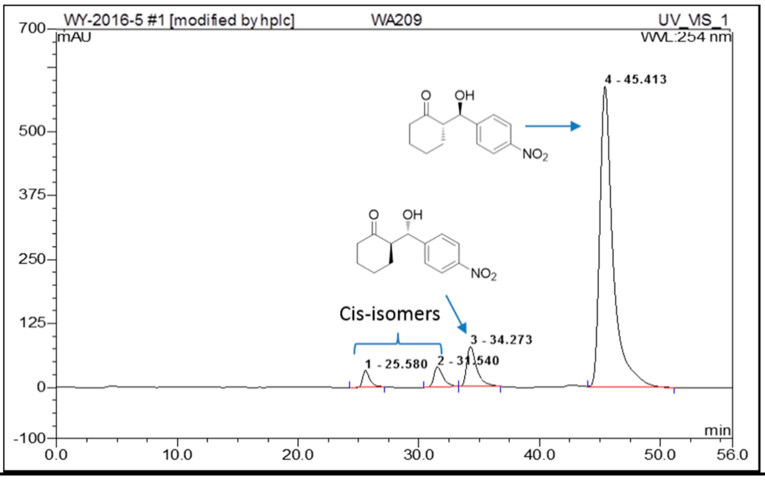

No.	Ret. Time/min	Peak Name	Height/mAU	Area/mAU*min	Rel. Area/%
1	21.91	1#	151.894	95.055	5.22
2	26.95	2#	159.086	132.068	7.25
3	29.34	3#	305.176	262.831	14.43
4	39.02	4#	1231.101	1331.268	73.10
**Total:**			1847.256	1821.222	100.00

Figure S8. A representative HPLC analysis of the aldol products. Mobile phase: *n*-hexane: *i*PrOH = 1:9, 0.8 mL/min.

Ref.:

1. Fineman, M.; Ross, S.D. Linear method for determining monomer reactivity ratios in copolymerization. *J. Polym. Sci.* **1950**, *5*, 259–262.
